# Single-session pelvic prehabilitation improves continence recovery after robot-assisted radical prostatectomy: a prospective comparative study

**DOI:** 10.1007/s11701-026-03608-x

**Published:** 2026-07-20

**Authors:** Luca Roggero, Riccardo Bertolo, Mattia Ronca, Elisabetta Muscolino, Martina Dolci, Sarah Malandra, Rossella Elia, Mauro Zatachetto, Paola Gaioni, Elisabetta Muraro, Federica Spiazzi, Paola Lamberti, Alessandro Veccia, Ermes Vedovi, Alessandro Antonelli

**Affiliations:** 1 University of Verona, Azienda Ospedaliera Universitaria Integrata, Borgo Trento Hospital, Verona, Italy; 2 Functional Recovery and Rehabilitation Unit, Department of Neurosciences, University Hospital of Verona, Verona, Italy; 3 Department of Urology, University of Verona, Azienda Ospedaliera Universitaria Integrata Verona, Piazzale Aristide Stefani, 1, Verona, 37126 Italy

**Keywords:** Prostate cancer, Prostatectomy, Urinary incontinence, Prehabilitation

## Abstract

Post-robot-assisted radical prostatectomy (RARP) urinary incontinence (PPI) represents the functional complication with the greatest negative impact on the patient’s global quality of life (QoL). Although the role of Pelvic Floor Muscle Training (PFMT) is widely established in perioperative clinical management, it is mostly delivered on demand after surgery. This study primarily assesses the clinical efficacy of pelvic prehabilitation when systematically implemented compared with usual care. This is a prospective, non-randomized, quasi-experimental (before-and-after) study comparing all consecutive patients undergoing RARP prior to the implementation of the PFMT program with an equally sized cohort of consecutive patients exposed to PFMT before RARP. PFMT consisted of a standardized 60-minute group session delivered by dedicated pelvic physiotherapists, including pelvic floor anatomy education, supervised proprioceptive and contraction exercises, and prescription of a structured home-based training program. The primary endpoint was post-operative continence, defined as no need for pads after surgery. Functional outcomes were also assessed using the UCLA-PCI and SF-36 questionnaires at 1, 3, and 6 months after surgery, and the recourse to post-operative PFMT. Additionally, a composite variable named “True Clinical Need” was defined as no incontinence at 6 months plus a moderate/severe subjective bother (score 3 or 4 on question Q5 of the UCLA-PCI). Finally, a cost-avoidance analysis on preoperative PFMT was performed. Overall, 214 consecutive subjects were recruited: 107 per our standard pathway (no-PFMT group) and 107 who received PFMT before RARP (PFMT group). Patient-reported satisfaction and adherence to the home-based program were high (both > 8/10 on a Likert scale). Demographic, oncological, and surgical features were comparable between the two groups. The PFMT group demonstrated significantly better 6-month continence rates (82.0% vs. 66.7%, *p* = 0.025) and urinary function scores (57.9 vs. 51.7, *p* = 0.045) compared with the NO-PFMT group. The recourse to individual postoperative cycles was similar between groups (no-PFMT 3.7% vs. PFMT 5.6%), as well the true clinical need (no-PFMT 16.1% vs. PFMT 11.2%). A cost-avoidance analysis showed that prehabilitation would yield an estimated net saving of €165 per patient. A single PFMT session could significantly improve continence recovery at 6 months after RARP within economic sustainability.

## Introduction

Despite the continuous technical and anatomical refinement in robot-assisted radical prostatectomy (RARP), post-prostatectomy urinary incontinence remains the complication with the most detrimental impact on patients’ overall quality of life (QoL). Although prevalence rates have improved over time, they remain clinically significant in some series [1, 2].

Within modern perioperative optimization pathways, pelvic floor rehabilitation - specifically pelvic floor muscle training (PFMT) - plays a well-recognized role in accelerating the recovery of striated sphincter function and compensating for the loss of proximal urethral resistance [3, 4].

Currently, the European Association of Urology guidelines strongly recommend PFMT for the conservative management of post-prostatectomy incontinence; however, the scientific literature remains divided on the optimal timing of the rehabilitative intervention [5].

Many standardized institutional protocols schedule the start of exercises only following the removal of the urinary catheter [6]. This timing, however, imposes the learning of complex motor patterns at a time when the local anatomy is already acutely altered by edema, neuropraxia resulting from dissection, and direct anastomotic trauma, potentially limiting the early efficacy of muscle recruitment [7]. Conversely, a growing body of evidence supports the rationale for preoperative prehabilitation. Several studies and recent meta-analyses suggest that initiating PFMT before surgery, when patients are free from pelvic pain and tissue inflammation, facilitates optimal acquisition of pelvic floor proprioception and significantly enhances early postoperative continence recovery [1, 8–10].

The present study aims to address this evidentiary gap by rigorously evaluating the concept of “prehabilitation” applied to PFMT. Specifically, it assesses outcomes following the implementation of a dedicated institutional protocol involving preoperative physiatric assessment and training prior to RARP.

The primary objective is to determine whether anatomo-functional education and the establishment of an anticipatory motor pattern before surgery can positively influence the trajectory of continence recovery and quality of life, through comparison with standard care. Additionally, the study seeks to quantify the economic impact of this preventive strategy on the healthcare system through a formal cost-avoidance analysis.

## Materials and methods

### Population and study design

To address whether the application of PFMT before RARP could be advantageous and convenient compared with a standard management protocol, implying recourse to PFMT only after surgery and on demand, we designed a pragmatic, prospective, non-randomized, quasi-experimental (before-and-after) study [11–13]. Accordingly, two size-matched cohorts were built: (1) the “PFMT” group included the patients who underwent the pelvic prehabilitation protocol prior to surgery; (2) the “NO-PFMT” group consisted of an equal number of the consecutive patients treated before the protocol implementation and did not receive preoperative pelvic prehabilitation. Ethical committee approval was obtained (4038CESC).

Baseline clinical, demographic, and pathological patient characteristics were prospectively collected, along with complete functional data recorded at baseline and at scheduled follow-up visits at 1, 3, and 6 months after surgery. Preoperative comorbidity risk and physical status were classified according to the American Society of Anesthesiologists (ASA) score. The latency elapsed between the preoperative physiotherapeutic session and surgery was codified as “lead time”. Urinary continence recovery was objectively assessed based on daily pad usage, with “continent” defined as no use of pads. The UCLA Prostate Cancer Index (UCLA-PCI), normalized on a 0-100 scale to isolate the Urinary Function and Urinary Bother domains, measured disease-specific functional recovery [14], while the Short-Form 36 (SF-36) was adopted to investigate global quality of life. The urinary functional domain was also investigated by recording the usage of post-operative rehabilitation courses and through a variable named “True Clinical Need”, defined as the presence at 6 months of objective incontinence (≥ 1 pad) with a moderate/severe subjective bother (score 3 or 4 on question Q5 of the UCLA-PCI). To evaluate perceived utility and adherence to the pre-operative PFMT program, a PREMs questionnaire with a 1–10 Likert scale was administered to each patient in this cohort. Additionally, sexual function recovery was longitudinally assessed via the International Index of Erectile Function (IIEF-5). Finally, an economic evaluation was conducted from the perspective of the Italian public healthcare system and was designed as a cost-avoidance analysis based on official regional reimbursement tariffs. The cost of the initial preventive group investment (Code 93.11.5: € 6.15) was contrasted with the financial burden of a formal individual cycle (Code 93.11.9: € 17.11 per session, for an estimated total of € 171.10 for 10 sessions). No indirect costs, patient-borne costs, quality-adjusted life years, or formal cost-effectiveness modelling were included.

### Pelvic pre-habilitation protocol

The preoperative PFMT was uniformly administered by dedicated pelvic physiotherapists according to a standardized protocol [4, 8]. The 60-minute group sessions (including a maximum of 6 patients) were conducted in a dedicated outpatient setting. The protocol began with an anatomo-physiological introduction using pelvic models to explain the surgical impact and potential complications (urinary incontinence, erectile dysfunction). This was followed by extensive counseling on behavioral modifications and lifestyle habits, including adequate hydration (1.5–2 L/day), physical activity, and optimal toilet posture (e.g., using a 20–25 cm footstool). Patients were strictly instructed to correct detrimental habits such as preventive voiding, straining during micturition, and the outdated “urine stop-test” [5, 15].

The core of the session focused on developing localized proprioception. Patients were instructed to sit on a hard surface, using a tennis ball placed at the central perineal tendon to maximize tactile feedback. Through deep perineal massage—achieved by shifting body weight antero-posteriorly and in circular motions—patients gained somatic awareness of the pelvic floor boundaries between the ischial tuberosities.

Following proprioceptive awakening, patients learned to cognitively and physically isolate the deep pelvic muscle plane. Activation involved contracting the anal sphincter while perceiving a retraction at the base of the penis, carefully avoiding parasitic contractions of synergistic muscles (glutes, adductors), while allowing the physiological co-activation of the transversus abdominis [16, 17]. Under continuous verbal coaching, patients performed supervised cycles of both phasic and tonic contractions. Phasic exercises consisted of rapid, maximal contractions to manage sudden intra-abdominal pressure peaks (e.g., coughing). Tonic exercises involved sub-maximal contractions sustained for 5–10 s, strictly followed by a rest period twice as long (10–20 s) to prevent muscle fatigue and restore resting continence.

To ensure continuity of care, patients were prescribed a daily Home-Based Program and provided with an informational booklet. The home prescription included one set of 50 rapid contractions and one set of 10 sustained contractions, to be performed two to three times a day, always preceded by the tennis ball massage. The goal of this rigorous training was to automate the guarding reflex—the anticipatory, voluntary contraction of the pelvic floor immediately prior to any effort that increases intra-abdominal pressure—thus preconditioning the neuromotor network before surgical trauma [16].

### Surgical technique and postoperative management

All surgical procedures were performed by expert surgeons (more than 500 RARP performed) using the DaVinci Xi surgical system (Intuitive Surgical, Sunnyvale, CA, USA), according to a conventional transperitoneal anterior approach [18, 19]. The indication for extended pelvic lymphadenectomy, the degree of prostatic dissection (including nerve-sparing) was performed according to International Guidelines [20] and the patient’s expectation. Whenever possible, a bladder neck-sparing technique and apical dissection prior to deep vascular complex ligation were adopted. Posterior reconstruction according to modified Rocco’s technique [21] and vesico-urethral anastomosis with double continuous suture were done in all cases [22].

Postoperative clinical management was standardized with early mobilization and resumption of oral feeding within the first postoperative day and discharge on day 3 or 4. Urethral catheter removal was routinely scheduled between postoperative days 10 and 12, primarily for logistical and organizational reasons.

### Statistical analysis

Descriptive statistics were used to summarize the baseline demographic, clinical, and pathological characteristics of the two cohorts. Continuous variables were reported as means and standard deviations (SD) or medians and interquartile ranges, depending on distributional normality. Sample size calculation was based on the difference in 6-month postoperative continence (“no safety pad”) reported by Rahota et al. (87.3% vs. 67.4%) [23]. Assuming a two-sided α error of 0.05 and a statistical power of 90%, at least 91 patients per group were required. To account for potential dropouts and missing follow-up data, the target enrolment was increased accordingly. Comparisons between groups for continuous variables were performed using the independent samples Student’s t-test or the Mann-Whitney U test, as appropriate. Categorical variables were expressed as frequencies and percentages, and differences between the groups were evaluated using Pearson’s chi-squared test or Fisher’s exact test, depending on the expected cell counts. To explore the relationship between the preoperative lead time and long-term functional outcomes, Spearman’s rank correlation coefficient was calculated. All statistical tests were two-sided, and p-values < 0.05 were considered statistically significant. Data analysis was performed in Python (Python Software Foundation, Wilmington, DE, USA) using the Pandas and SciPy libraries for data manipulation and statistical computation.

## Results

### Primary analysis: objective continence and functional impact

**Fig. 1:**
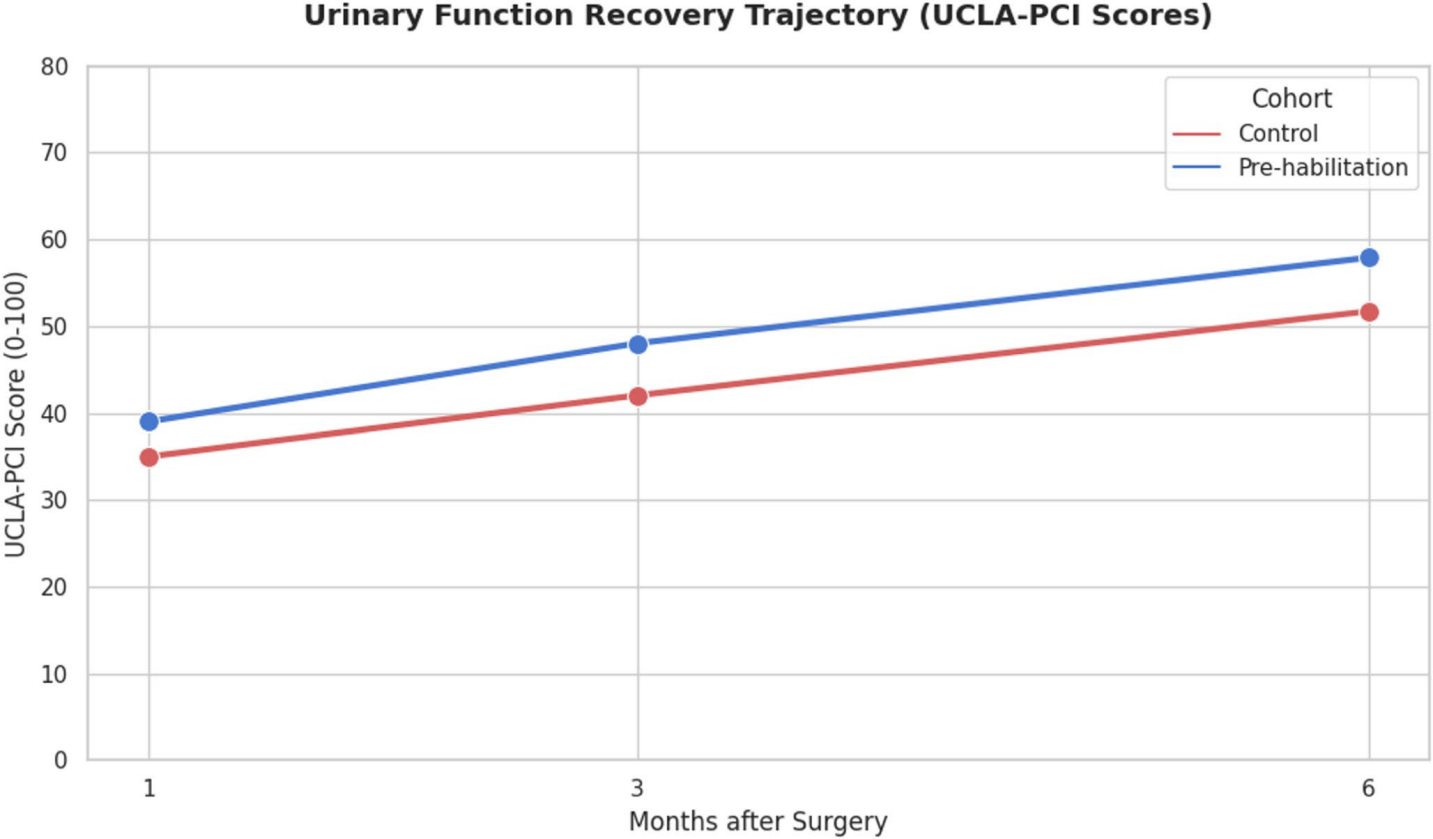
Objective continence recovery after robot-assisted radical prostatectomy according to prehabilitation status. Bar chart showing the proportion of patients achieving objective continence after surgery in the control and prehabilitation cohorts. Objective continence was defined as no pad use per day. Patients who underwent preoperative pelvic floor muscle prehabilitation showed a higher continence recovery rate compared with those managed with usual care

A total of 214 patients were included in the analysis, with 107 in each group. The comparison of all baseline features showed that the two cohorts were well balanced (Table 1), including the rate of nerve-sparing surgery, which was comparable between groups (72.0% in the NO-PFMT group vs. 74.0% in the PFMT group, *p* = 0.7). The analysis of the primary endpoint showed a positive impact of preoperative PFMT across all follow-up time points. At 1-month post-surgery, the objective continence rate (0 pad/day) was 51.1% (48/94) in the pre-habilitated cohort versus 46.6% (41/88) in the control cohort (*p* = 0.6) (Table 2). At 3-months, continence rates reached 74.2% (69/93) and 60% (51/85), respectively, approaching statistical significance (*p* = 0.063). The positive trajectory culminated at 6 months, where the prehabilitated cohort recorded an objective continence rate of 82% (73/89), significantly higher than the 66.7% (58/87) observed in the non-prehabilitated cohort (*p* = 0.03) (Table 3; Fig. 1).

**Table 1:** Baseline characteristics and preoperative clinical data. Comparison of demographic, clinical, anesthesiologic, biopsy-related, and surgical baseline characteristics between patients who did not receive preoperative physiotherapy and those who underwent preoperative pelvic floor muscle prehabilitation before robot-assisted radical prostatectomy. Continuous variables are reported as mean ± standard deviation, whereas categorical variables are reported as percentages or number of patients. BMI: body mass index; ASA: American Society of Anesthesiologists; ISUP: International Society of Urological Pathology

Preoperative Parameter	NO PFMT preop. (*N* = 107)	PFMT preop. (*N* = 107)	*p*-value
Mean Age (years ± SD)	66.1 ± 6.2	65.8 ± 5.8	0.7
Mean BMI (kg/m² ± SD)	26.3 ± 3.4	25.5 ± 3.1	0.5
Prostate Volume (ml ± SD)	47.5 ± 12.4	41.9 ± 11.8	0.4
ASA Score, n (%)	(*N* = 101)	(*N* = 95)	0.3
ASA I	5 (5.0%)	6 (6.3%)	
ASA II	73 (72.3%)	76 (80.0%)	
ASA III	23 (22.8%)	13 (13.7%)	
Biopsy ISUP Grade Group (%)			0.9
- ISUP 1	20.2%	22.0%	
- ISUP 2	43.0%	47.0%	
- ISUP 3	25.9%	24.2%	
- ISUP 4–5	10.9%	6.8%	
Nerve-Sparing Surgery (Bi/Uni)	72.0%	74.0%	0.7

**Table 2:** Perioperative data and oncological follow-up. Comparison of perioperative outcomes, definitive pathological findings, surgical margin status, postoperative prostate-specific antigen levels, and recurrence or postoperative treatment rates between the no-prehabilitation and prehabilitation cohorts. Continuous variables are reported as mean ± standard deviation when applicable, and categorical variables are reported as percentages or number of patients. PSA: prostate-specific antigen; ISUP: International Society of Urological Pathology

Oncological Parameter	NO PFMT preop. (*N* = 107)	PFMT preop. (*N* = 107)	*p*-value
Length of Stay (mean days ± SD)	4.48 ± 1.39	4.53 ± 1.07	0.7
Definitive Pathological ISUP (%)			0.4
- ISUP 1	8.9%	12.2%	
- ISUP 2	50.3%	47.3%	
- ISUP 3	23.6%	29.8%	
- ISUP 4–5	17.2%	10.7%	
Pathological Stage (pT), n (%)			1
pT2 (Localized disease)	85 (79.4%)	84 (79.2%)	
pT3/pT4 (Locally advanced)	22 (20.6%)	22 (20.8%)	
Positive Surgical Margins (Yes)	24.1%	19.0%	0.5
Postoperative PSA (mean, ng/ml)	0.07	0.09	0.3
Recurrence / Post-op Therapy	4.2%	4.2%	1

**Table 3:** Functional outcomes and objective continence recovery during follow-up. Longitudinal comparison of objective continence, urinary function, urinary bother, and sexual function between patients with and without preoperative pelvic floor muscle prehabilitation at 1, 3, and 6 months after robot-assisted radical prostatectomy. Objective continence was defined as the use of no pads per day. Urinary function and urinary bother were assessed using the UCLA Prostate Cancer Index, while sexual function was assessed using the International Index of Erectile Function-5. Values are reported as n/N (%) or mean ± standard deviation. UCLA-PCI: University of California, Los Angeles Prostate Cancer Index; IIEF-5: International Index of Erectile Function-5

Outcome Measure	Time Point	NO PFMT preop. (*N* = 107)	PFMT preop. (*N* = 107)	*p*-value
**Objective Continence**	1 Month	41/88 (46.6%)	48/94 (51.1%)	0.6
(0 pad/day), n/N (%)	3 Months	51/85 (60.0%)	69/93 (74.2%)	0.055
	**6 Months**	**58/87 (66.7%)**	**73/89 (82.0%)**	0.025*
**Urinary Function**	1 Month	48.2 ± 18.5	50.1 ± 19.2	0.5
(UCLA-PCI), mean ± SD	3 Months	49.8 ± 20.1	53.4 ± 19.8	0.2
	**6 Months**	**51.7 ± 22.4**	**57.9 ± 21.8**	0.045*
**Urinary Bother**	1 Month	62.5 ± 24.1	64.8 ± 23.5	0.8
(UCLA-PCI), mean ± SD	3 Months	68.2 ± 25.6	72.4 ± 24.8	0.7
	**6 Months**	**72.3 ± 25.1**	**79.1 ± 23.5**	**0.2**
**Sexual Function**	1 Month	3.9 ± 4.6	3.4 ± 5.5	0.7
(IIEF-5), mean ± SD	3 Months	4.2 ± 5.0	4.2 ± 5.4	1
	**6 Months**	**4.8 ± 5.6**	**5.4 ± 6.8**	**0.6**

**Fig. 2:**
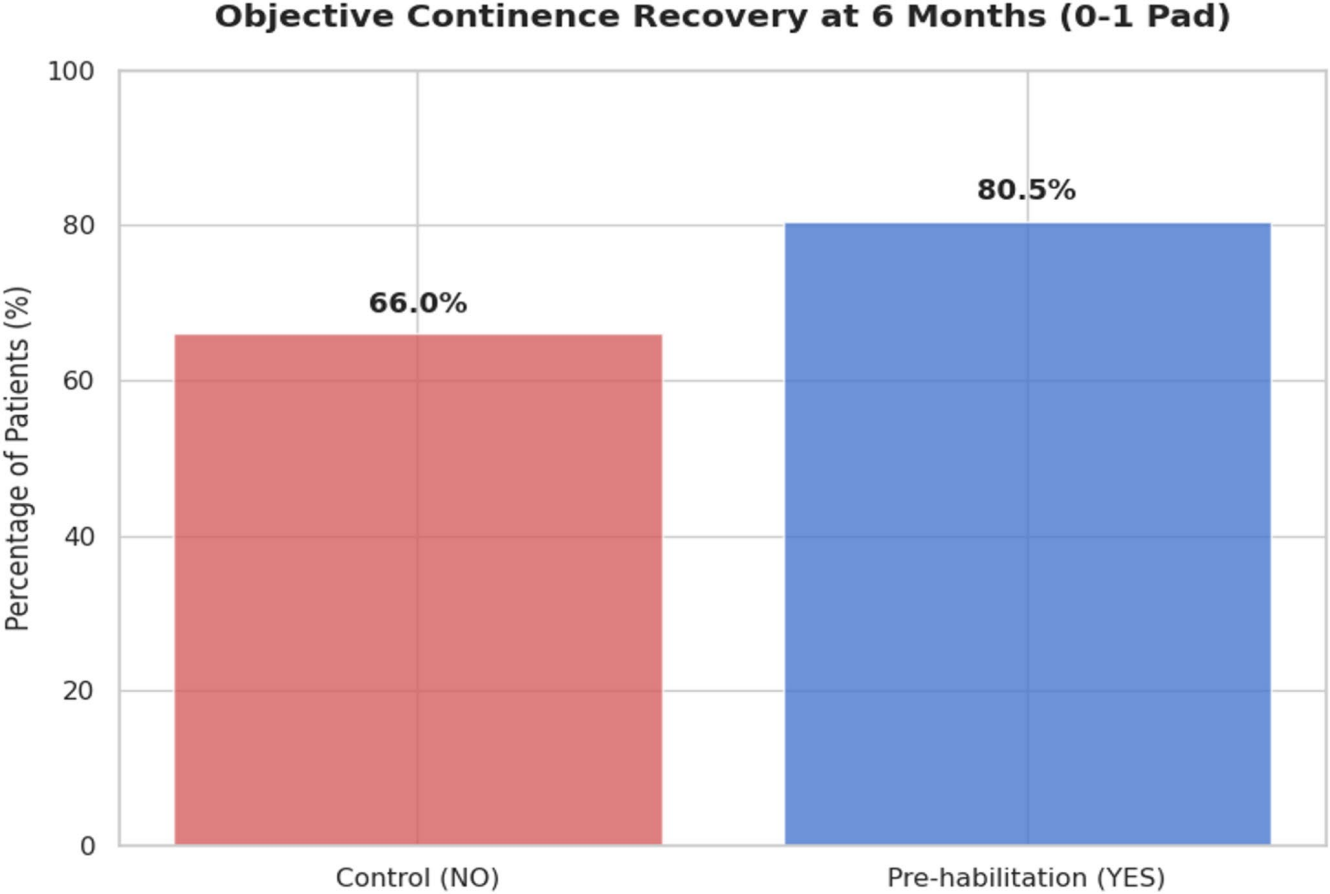
Urinary function recovery trajectory after robot-assisted radical prostatectomy. Line chart showing longitudinal changes in UCLA Prostate Cancer Index urinary function scores at 1, 3, and 6 months after surgery in the control and prehabilitation cohorts. Higher scores indicate better urinary function. Patients receiving preoperative pelvic floor muscle prehabilitation demonstrated a more favorable recovery trajectory over time

This functional superiority was consistently reflected in the Urinary Function domain of the UCLA-PCI. The prehabilitated cohort recorded higher mean scores, achieving a significantly higher functional index at 6 months compared to the unexposed group (57.9 vs. 51.7, *p* = 0.045) (Fig. 2).

Regarding the global perception of quality of life (SF-36), physical functionality (Physical Functioning) at 1 month showed a trend in favor of the prehabilitated patients (81.7 vs. 76.0, *p* = 0.082). Concurrently, the General Health domain demonstrated progressive improvement, resulting in significantly superior and consolidated outcomes in the prehabilitated cohort at 6 months (49.6 vs. 46.8, *p* = 0.03).

Finally, sexual function was explored using the IIEF-5 questionnaire. Mean scores remained low in both groups at 6 months (5.4 ± 6.8 in the PFMT group vs. 4.8 ± 5.6 in the NO-PFMT group). These findings should be interpreted descriptively given the limited discriminatory capacity of the instrument at such low score levels.

### Perioperative data and oncological follow-up

The analysis of perioperative data and oncological follow-up confirmed the homogeneity of the two cohorts. All evaluated oncological outcomes and surgical parameters were comparable between the groups (Table 2).

### Secondary analysis: preoperative lead time and recourse to structured PFMT

**Fig. 3:**
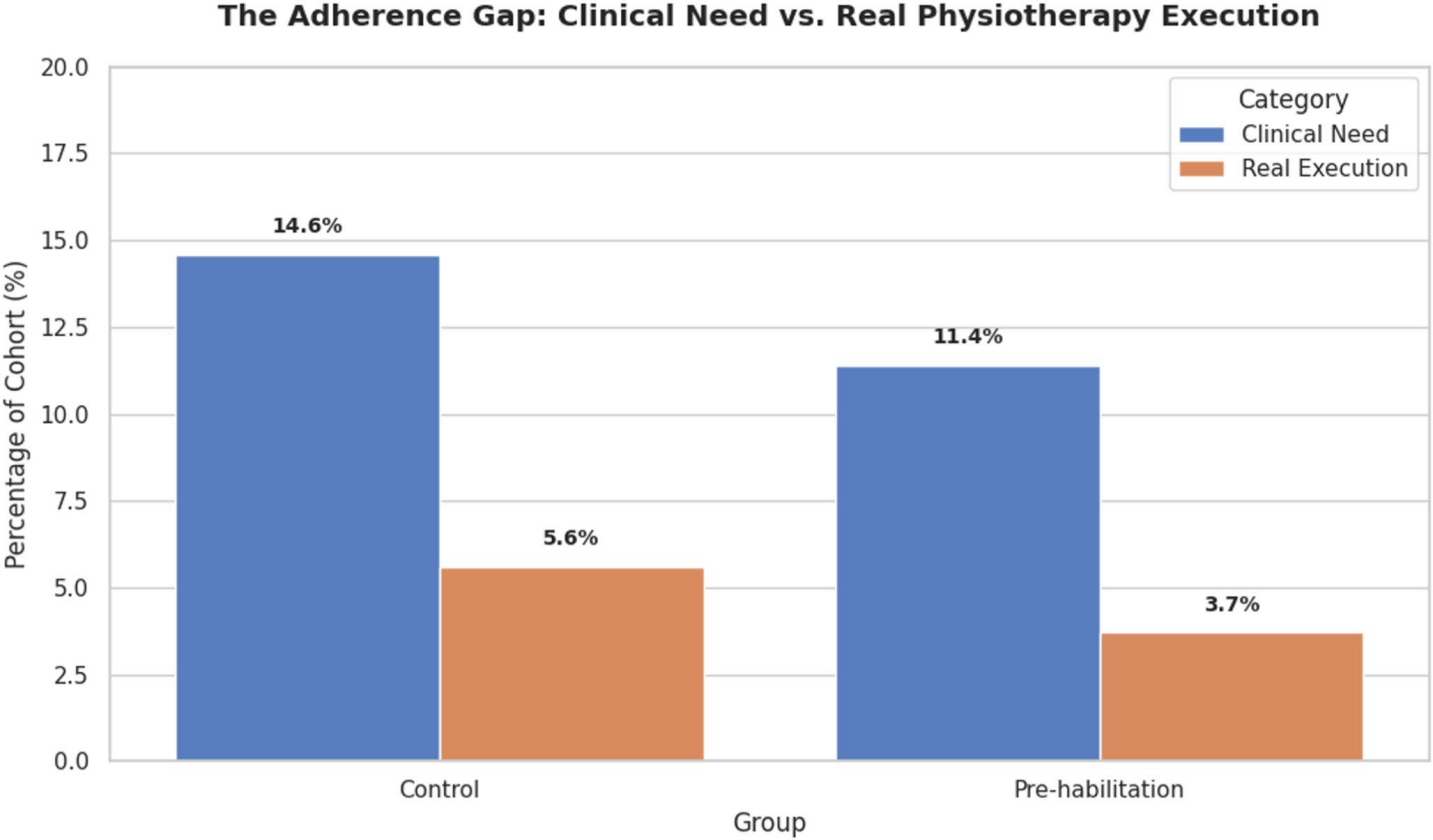
Adherence gap between the clinical need for postoperative physiotherapy and its actual delivery. Grouped bar chart comparing the estimated true clinical need for postoperative pelvic floor muscle training with the proportion of patients who actually underwent structured postoperative physiotherapy in the control and prehabilitation cohorts. The figure highlights the discrepancy between rehabilitation need and real-world treatment uptake, as well as the lower clinical need observed after preoperative prehabilitation

In the prospective intervention cohort, the preoperative physiotherapeutic session was administered with a median lead time of 21 days (interquartile range: 11–29 days; overall range: 1–98 days) prior to the surgical procedure. Spearman’s correlation analysis revealed no statistical association between this temporal interval and functional outcomes at 6 months (all p-values > 0.25). Regarding the post-surgical rehabilitative burden, in the non-prehabilitated cohort, 16.1% of patients had a clear clinical need for postoperative physiotherapy, compared with 11.2% in the prehabilitated cohort (*p* = 0.4). Consequently, actual recourse to the individual cycle was 5.6% (6/107) in the control group and was limited to 3.7% (4/107) in the intervention group (*p* = 0.7) (Table 4; Fig. 3).

**Table 4:** Secondary analysis of logistics, pelvic floor muscle training adherence, and cost avoidance. Comparison of secondary endpoints related to preoperative lead time, correlation between lead time and functional outcomes, true clinical need for postoperative pelvic floor muscle training, actual postoperative physiotherapy execution, home-program adherence, patient-reported satisfaction, and estimated cost avoidance. Patient-reported adherence and satisfaction were assessed using Likert-scale PREMs. PFMT: pelvic floor muscle training; PREMs: patient-reported experience measures; n.s.: not significant

Secondary Endpoints	NO PFMT preop. (*N* = 107)	PFMT preop. (*N* = 107)	*p*-value
Lead Time (days, median and range)	--	14.2 (2–45)	--
Lead Time / Outcome Correlation	--	r = n.s.	> 0.25
True Clinical Need for PFMT (%)	16.1% (14/87)	11.2% (10/89)	0.4
Actual PFMT Executed (post-op)	5.6% (6/107)	3.7% (4/107)	0.7
Home Program Adherence	--	8.2 / 10	--
General Satisfaction (PREMs)	--	9.0 / 10	--
Net Savings (Cost Avoidance)	--	€ 164.95	--

### Organizational sustainability, economic analysis, and PREMs

Against a minimal preventive group investment (€6.15 per patient), the system systematically avoids the costs associated with early therapeutic failure. Based on the assumptions of the model, prehabilitation was associated with an estimated potential cost avoidance of approximately €165 per patient (Table 4). Protocol acceptance (PREMs) was excellent, with a General Satisfaction score of 9/10 and home adherence > 8.2/10.

## Discussion

The present study provides additional evidence supporting the role of preoperative PFMT as a key modulator of functional recovery after RARP. In particular, our findings suggest that even a single-session, structured prehabilitation intervention is associated with improved continence recovery at 6 months, together with a measurable reduction in the need for postoperative rehabilitation and favorable cost implications.

These results are consistent with and extend prior research on prehabilitation in the RARP setting. In a prospective comparative study, Rahota et al. reported that a structured 1-day multidisciplinary prehabilitation program significantly improved continence recovery both at 1 month (60% vs. 37%) and 6 months (87.3% vs. 67.4%), with prehabilitation emerging as an independent predictor of functional recovery [23].

Similarly, our data demonstrate a significant advantage at 6 months (82.0% vs. 66.7%), confirming the robustness of the effect even when the intervention is simplified to a single neuromotor-focused session.

Notably, while Rahota et al. adopted a more complex and resource-intensive multidisciplinary model, our protocol achieves comparable functional benefits with a markedly reduced organizational burden, suggesting that the key driver of efficacy may lie in early proprioceptive training and motor pattern acquisition rather than in program complexity per se.

More recently, Buhas et al. evaluated the impact of prehabilitation within a modern perioperative pathway, including digital delivery modalities, demonstrating improved early continence rates (84.2% vs. 67.6% at 6 weeks) and sustained benefits up to 12 months [24].

Importantly, their multivariable analysis confirmed prehabilitation as an independent protective factor against early incontinence. Our findings align with this evidence, particularly in supporting the concept that preoperative conditioning modifies the trajectory of continence recovery. However, our study differs in that it isolates the effect of a single-session intervention delivered in a pre-admission setting, without reliance on continuous digital engagement or prolonged training. This distinction is clinically relevant, as it demonstrates that meaningful functional gains can be achieved even in healthcare systems where adherence to long-term or technology-based programs may be limited.

From a broader perioperative perspective, integrating prehabilitation into enhanced recovery pathways has also been associated with improvements in surgical efficiency and healthcare costs.

Ploussard et al. reported that the combination of enhanced recovery after surgery (ERAS) and prehabilitation significantly reduced hospital stay (from 4.7 to 1.6 days) and overall costs, resulting in a total cost reduction of up to 22% compared with standard care [25]. Although our study did not find differences in perioperative metrics such as length of stay or operative time, the economic analysis provides complementary evidence. Specifically, our cost-avoidance model suggests that a minimal upfront investment (€6.15 per patient) may reduce the need for structured postoperative rehabilitation cycles, corresponding to an estimated potential cost avoidance of approximately €165 per patient. While simplified, this finding is directionally consistent with the economic benefits reported in integrated ERAS–prehabilitation pathways and reinforces the sustainability of prehabilitation strategies even when implemented in a streamlined format.

A further clinically relevant aspect emerging from the comparison across studies is the issue of adherence. Both Rahota et al. and Buhas et al. highlight the importance of patient engagement, with digital solutions significantly increasing program uptake (up to ~ 69% in the latter).

In contrast, real-world adherence to postoperative physiotherapy remains suboptimal, often limited by logistical and psychological barriers [26]. Our data confirm this gap, showing a discrepancy between clinical need and actual access to postoperative rehabilitation. In this context, prehabilitation may represent a pragmatic strategy to “front-load” physiotherapeutic education at a time when patients are more motivated and physically able to learn, thereby mitigating the impact of poor postoperative adherence.

From a mechanistic standpoint, the consistency of findings across these studies supports the hypothesis that prehabilitation acts primarily through early acquisition of pelvic floor proprioception and anticipatory motor control.

Interestingly, no association was observed between the preoperative lead time and continence recovery. This finding may be explained by the nature of the intervention itself, which was primarily conceived as an educational and neuromotor learning strategy rather than a prolonged conditioning program. The acquisition of pelvic floor awareness, correct muscle recruitment, and behavioral recommendations may therefore be beneficial even when delivered shortly before surgery, provided that patients are given the opportunity to continue the prescribed home-based exercises.

Training patients before surgery allows them to internalize the guarding reflex in a physiologically intact environment, which may facilitate more effective muscle recruitment after surgical trauma. This concept is coherent with experimental evidence on pelvic floor activation patterns and provides a plausible explanation for the observed improvements in early and mid-term continence recovery.

In recent years, considerable efforts have been directed towards defining “best practice” strategies to optimize functional outcomes after RARP, with a strong focus on surgical planning, anatomical preservation techniques, and intraoperative decision-making. A recent European consensus statement highlighted the central role of preoperative surgical planning in improving oncological and functional outcomes, emphasizing factors such as nerve-sparing strategies, urethral preservation, and imaging-based risk stratification [27].

However, this predominantly surgeon-centered perspective may overlook simpler, patient-directed interventions that can meaningfully influence recovery trajectories. In this context, prehabilitation represents a low-cost, low-complexity strategy that acts upstream of surgical trauma, yet remains underrepresented in standardized care pathways. Our findings, together with emerging evidence, suggest that early neuromotor conditioning may complement technical refinements and contribute independently to continence recovery.

First, the non-randomized design may introduce selection bias and residual confounding into the analysis. However, the intervention was implemented in a consecutive, unselected cohort once the protocol was introduced into routine clinical practice, rather than being offered to a pre-defined subgroup. This pragmatic “real-world” design is reflected in the comparable baseline clinical, oncological, and surgical characteristics observed across groups, suggesting minimal selection bias and reducing the need for complex statistical adjustments, such as propensity score matching.

Second, all procedures were performed by highly experienced surgeons beyond their learning curve, within a high-volume setting. As recently demonstrated by our group, high surgical volume and experience can significantly overcome the impact of adverse prostatic-urethral anatomy on continence recovery, ensuring a standardized, high-quality functional baseline for all patients in both cohorts [19]. While this may limit the generalizability of the findings to lower-volume centres, it also represents a strength, as the observed benefit of prehabilitation emerges in a context of already optimized surgical performance and functional outcomes, which are often superior to those reported in the broader literature. In this sense, the added value of prehabilitation appears to be incremental and independent from surgical expertise. Catheter removal was routinely performed between postoperative days 10 and 12, which is later than in many contemporary RARP series. Although this institutional practice was applied uniformly across both cohorts, it may have influenced the assessment of very early continence recovery and should be considered when comparing our results with those of other studies.

Third, the definition of continence based on pad usage, although widely adopted in the literature, may be subject to reporting variability and may not fully capture patients’ perceptions of urinary function.

Fourth, the clinical interpretation of the observed difference in UCLA-PCI Urinary Function scores should be made cautiously, as no universally accepted MCID has been established for this instrument in the post-prostatectomy setting. Nevertheless, the improvement in patient-reported urinary function was accompanied by a concordant increase in objectively measured continence rates. Moreover, interpretation of the sexual function analyses is further limited by the very low postoperative IIEF-5 scores observed in both cohorts, which may have introduced a floor effect and reduced the ability to detect meaningful between-group differences.

Fifth, an additional limitation is the progressive loss to follow-up over time. Some patients declined to participate in the scheduled postoperative assessments and therefore did not provide complete questionnaire data, reducing the number of evaluable subjects at 6 months and potentially introducing attrition bias.

Finally, the economic evaluation is based on a simplified cost-avoidance model and does not account for indirect costs, patient-borne expenses, or variability across healthcare systems; it should therefore be interpreted as an estimate of potential economic impact rather than a formal cost-effectiveness analysis. Moreover, the model assumes completion of a full postoperative rehabilitation cycle in patients with a clinical need for treatment and therefore estimates potential rather than realized healthcare savings.

## Conclusions

A single-session group prehabilitation program in the pre-admission phase is associated with superior functional outcomes, organizational flexibility, and tangible economic advantages. By significantly improving objective continence at 6 months (82.0%) and reducing the dependence on postoperative rehabilitative cycles—which are traditionally hindered by low patient adherence—prehabilitation represents a pragmatic and sustainable strategy to optimize post-prostatectomy quality of life and healthcare resource allocation.

Future longitudinal studies are needed to determine whether the improvement observed at 6 months is sustained over time or gradually mitigated as continence recovery approaches its natural plateau.

## Data Availability

No datasets were generated or analysed during the current study.

## Declarations

### Permission to reproduce material from other sources

N/A.

### Clinical trial registration

N/A.
